# Prognostic implication of isolated pulmonary nodules in patients with a history of breast cancer

**DOI:** 10.1186/s13019-022-01898-4

**Published:** 2022-06-18

**Authors:** Weigang Zhao, Chuanli Song, Shu Zhu, Zuodong Song

**Affiliations:** 1 Department of Thoracic Surgery, Shanghai Jiao Tong University Affiliated Sixth People’s Hospital, Shanghai, 200233 China; 2 Department of Thoracic Surgery, Jimo District Qingdao Hospital of Traditional Chinese Medicine, Shandong, 266200 China; 3 Department of Ultrasound, Shanghai Sixth People’s Hospital Affiliated to Shanghai Jiao Tong University, Shanghai, 200233 China; 4 Shanghai Lung Cancer Center, Shanghai Chest Hospital, Shanghai Jiao Tong University, Shanghai, 200030 China

**Keywords:** Pulmonary nodules, Lung cancer, Breast cancer, Surgery

## Abstract

**Background:**

Isolated malignant pulmonary nodules were frequently seen in patients with breast cancer. These were metastasis from the breast cancer or new primary lung cancer. The role of surgery for such pulmonary nodules remains unclear.

**Methods:**

A total of 90 patients who underwent surgery for solitary malignant pulmonary nodules between January 2010 and April 2018 after curative operation for breast cancer were reviewed.

**Results:**

The pathologic diagnoses revealed 63 patients with primary lung cancer (PLC) and 27 patients with pulmonary metastatic breast cancer (MBC), which were divided into two groups. All patients were female with a mean age of 55.08 ± 9.84 years (range 31–75). Age differences between the two groups were insignificant. Of the 63 patients with PLC, 55(87%) had a lobectomy with lymphadenctomy and 8(13%) had a limited resection, while the majority of patients (78%) with MBC had a limited resection. All nodules were adenocarcinomas and their mean diameter was 1.63 ± 0.57 cm. 7/55 of patients with PLC had N1 disease while 3/6 of those with MBC had involvement of N1 nodes. For all patients, the overall survival (OS) was 86.1% at 5 years and the disease-free survival (DFS) was 86.0% at 5 years. Patients with PLC had the better surgical outcomes including OS and DFS than those with MBC did (94.2% vs. 72.8%, *p* = 0.017; 93.6% vs. 63.9%, *p* = 0.002).

**Conclusions:**

Surgical outcomes of isolated malignant pulmonary nodules in breast cancer patients were favorable. Surgery should be considered as an option for breast cancer patients with isolated pulmonary nodules.

## Introduction

Synchronous or metachronous malignant lung nodules are seen with increasing frequency in patients with breast cancer. And essentially, these are metastasis from the breast cancer or new primary lung neoplasms. This increased incidence relates to better follow up clinics, routine use of computed tomography (CT) or even positron emission tomography (PET) scanning during follow up and increased frequency of small lung adenocarcinomas. In a 2012 review [1], the incidence of metastatic lesions varied from 34 to 75%, that of primary lung cancer from 12 to 48%, and that of benign lesions from 14 to 18%.

Although surgery is nearly always indicated for these nodules, the type of resectional procedure may vary from limited resections in cases of solitary metastases to extended resections with lymphadenectomy if the lesion is a primary lung cancer [2]. More importantly, little information is available on outcomes after surgical resection.

The aim of this retrospective study was to evaluate the role of surgery in the management of malignant solitary pulmonary nodules in patients with breast cancer as well as to determine their prognosis based on the histopathological nature of the lesion.

## Material and methods

This study was approved by the Institutional Review Board at the Shanghai Chest Hospital. The requirement for patient consent was waived. From January 2010 to April 2018, breast cancer patients who underwent surgical resection of isolated neoplastic pulmonary nodules defined as cancer nodules with a diameter of 3 cm or less and surrounded by normal lung parenchyma were included in this study. Patients with nodules with a diameter greater than 3 cm as well as those with more than one nodule and those with satellite nodules were excluded from study. Patients with documented other sites of metastatic disease of their breast cancer were excluded as well.

Preoperative, operative, and pathological data were collected by review of medical records. Preoperative staging typically included CT of the chest and abdomen while PET was not performed routinely. For each case, histological analysis using morphology and thyroid transcription factor-1 (TTF-1) was used to differentiate between primary lung cancer (PLC) and metastatic disease from breast cancer (MBC) [3].

Patients were followed at our outpatient clinic with standard chest radiographs and often CT scanning or by telephone contact. If tumor recurrence was suspected, pathological confirmation was usually obtained.

The end-points of the study were DFS which was measured from the date of surgery until the date of first recurrence defined either as proven metastasis or CT consistent with metastatic disease and OS which was measured from the date of operation until the date of death from all causes or the date last seen alive.

A comparison between the histopathological characteristics of the resected lesions (PLC vs MBC) and surgical outcome was obtained.

### Statistics

All of the data were analyzed with SPSS 16.0 (SPSS Inc., Chicago, IL). The Pearson chi-square test and Fisher’s exact test were used to determine the statistical significance of each of the categorical variables. Student t-test was used to compare the mean values of the numerical variables. The survival curves of DFS and OS were estimated by the Kaplan–Meier method. Differences in survival between the two groups were assessed by the log-rank test. *P* values were 2 tailed for all the tests. *P* < 0.05 was considered statistically significant.

## Results

### Patients demographics

A total of 153 breast cancer patients with pulmonary lesions detected by thoracic CT or PET/CT scan received surgical resection at department of thoracic surgery, Shanghai chest hospital, Shanghai Jiao Tong University. Twenty patients with pathologically confirmed benign lung diseases and 25 patients with multiple pulmonary lesions were excluded. Eighteen patients with pulmonary nodules > 3 cm were excluded as well. Totally, 90 breast cancer patients with isolated malignant pulmonary nodules were enrolled in this study. The 90 patients were divided in one of two groups, group 1 (n = 63) being those with primary lung cancer (PLC) and group 2 (n = 27) those with a metastasis from their breast cancer (MBC). Patient clinical characteristics were summarized in Table 1. Age, gender and interval between the treatment of breast cancer and the occurrence of the lung nodule (Disease-free Interval, DFI) were documented. All patients were female with a mean age of 55.08 ± 9.84 years (range 31–75). There was no significant age difference between patients with PLC (mean age of 55.81 ± 8.94) and those with MBC (mean age of 53.37 ± 11.67). In 11 patients, the lung nodule was synchronous with that of the breast cancer and in 79, it was metachronous. The average DFI was 1.50 ± 0.5 months in PLC group and 6.25 ± 443.48 months in MBC group (*p* < 0.001). The pulmonary nodules were located on the same side of breast cancer in 31 cases in PLC group and 16 cases in MBC group. Higher rate of stage II and III breast cancers were detected in MBC group (*p* = 0.003).

**Table 1:** Clinical characteristic of 90 patients with malignant pulmonary nodules

characteristic	PLC group	MBC group	P value
Gender			N/A
Female	63 (100.0%)	27 (100.0%)	
Age (years)	55.81 ± 8.94	53.37 ± 11.67	0.263
DFI (months)	1.50 ± 0.50	56.25 ± 43.48	< 0.001
Location Ipsilatera Contralateral	31 32	16 11	0.382
pStage of breast cancer stage I stage II stage III	25 (39.7%) 29 (46.0%) 9 (14.3%)	1 (3.7%) 20 (74.1%) 6 (22.2%)	0.003

*PLC* primary lung cancer, *MBC* metastatic breast cancer, *DFI* disease-free interval

Extent of pulmonary resection and pathological characteristics of the resected nodules are summarized in Table 2. Size of the lesions, histology and presence or absences of metastatic lymph nodes were documented. Of the 63 patients with PLC, 55(87%) had a lobectomy with mediastinal lymph node dissection and 8(13%) had a limited resection, mostly because the exact nature of the nodule could not be determined intraoperatively. By contrast, the majority of patients (78%) with MBC had a limited resection, usually without nodal sampling. All nodules were adenocarcinomas and their mean diameter was 1.63 ± 0.57 cm. In patients who had intraoperative nodal staging, 7/55 of those with PLC had N1 disease while 3/6(50%) of those with MBC had involvement of either the bronchopulmonary or mediastinal nodes.

**Table 2:** Perioperative results and pathological characteristics between PLC and MBC group

characteristic	PLC group	MBC group	P value
Extent of resection			N/A
Limited resection	8 (12.7%)	21 (77.8%)	< 0.001
Lobectomy	55 (87.3%)	6 (22.2%)	
Size (cm)	1.63 ± 0.69	1.57 ± 0.72	0.691
pN stage of patients received LND N0 N1	48 7	3 3	0.019

*PLC* primary lung cancer, *MBC,* metastatic breast cancer, *LND* lymph node dissection

### Overall and disease free survival after surgery

**Fig. 1:**
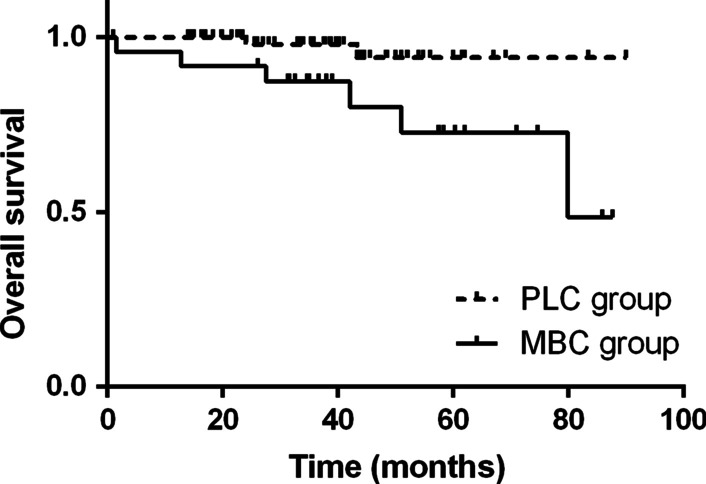
Kaplan–Meier curves for overall survival in 90 patients who underwent surgery for solitary malignant pulmonary nodules

Follow-up ranged from 0 to 90 months (median, 37.9 months). For the entire group, the overall survival (OS) was 86.1% at 5 years and the mean survival was 80.71 ± 3.05 months (95% CI: 74.73–86.69).Overall survival was significantly better in patients with PLC than in those with MBC (94.2% versus 72.8%, *p* = 0.017) (Fig. 1). Similarly, mean survival was better in patients with PLC being 86.89 ± 2.16 months (95% CI: 82.66–91.12) versus 70.48 ± 5.98 months (95% CI: 58.76–82.20) for those with MBC.

**Fig. 2:**
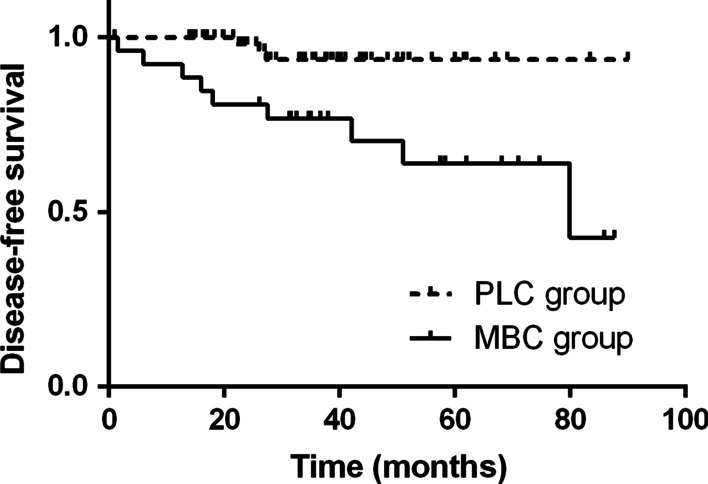
Kaplan–Meier curves for disease-free survival in 90 patients who underwent surgery for solitary malignant pulmonary nodules

For the entire group, overall disease free survival (DFS) was 86.0% at 5 years and mean DFS was 77.38 ± 3.35 months (95% CI: 70.80–83.95). Like observed in overall survival, DFS was significantly better in patients with PLC than in those with MBC (93.6% vs. 63.9%, p = 0.002) (Fig. 2). Mean DFS was 85.87 ± 2.31 months (95% CI: 81.35–90.40) for patients with PLC and 63.57 ± 6.47 months (95% CI: 50.87–76.25) for those with MBC.

## Discussion

Pulmonary is one of the most common sites for breast cancer metastasis. The majority of pulmonary nodules in breast cancer patients are pulmonary metastases [1]. However, the prevalence of primary lung cancer following breast cancer is significant, which accounts for about 5% of second primary malignancies among breast cancer survivors [4]. In this study, the prevalence of primary lung cancer was 56.2% (86 patients with primary lung cancer in all 153 patients), while the prevalence of pulmonary metastases was 30.7% (47/153). The underlying factors increasing the risk of primary lung cancer arising in breast cancer patients might be the improved outcomes for breast cancer and the long-term complications of anticancer treatment [5]. Breast cancer patients always comply with the regular follow-up after mastectomy. Accordingly, a high percentage of small adenocarcinomas were found in this study, which promises a favorable prognosis. Surgical treatment is always advocated for early-staged non-small-cell lung cancers (NSCLCs). Patients with early-staged disease receiving VATS lobectomy have a 5-year survival rate of 80%-90% [6]. According to our results, breast cancer patients with PLC had a mean OS time of 86.89 ± 2.16 months and a 5-year survival rate of 94.2%, which was exciting. Notably, besides a high percentage of stage IA NSCLCs (88.9%; 56/63) was revealed, there were 7 unexpected microscopic N1 cases in this study (11.1%; 7/63). Although patients with small-sized NSCLCs were considered as low-risk patients, the overall rate of nodal upstaging was not very low, approaching nearly 16% [7]. But small-sized tumors with incidental N1 diseases always have a good prognosis. It could be treated with surgical resection without induction therapy [8, 9].

The role of surgery for pulmonary metastasis of breast cancer is unclear in the era of multimodality therapy. Breast cancer with pulmonary metastasis is usually considered as a systemic disease. More effective systemic chemotherapies including anti-HER2 treatment are usually applied, thus the prognosis of metastatic breast cancer patients has been greatly improved. However, some clinical studies have suggested that surgical resection of pulmonary metastases could achieve the satisfactory outcomes [10, 11]. In 1992, Staren et al. reported a 5-year survival rate of 36% for metastatic breast cancer patients receiving pulmonary resection. They indicated a potential survival benefit from surgical treatment compared with medical treatment only for such patients [12]. In 2003, C. Ludwig et al. reported that the median survival time and median DFS after resection of lung metastases for breast cancer patients were 96.9 months and 28.8 months respectively for isolated pulmonary metastasis [13]. More recently, in 2005 Tanaka and his colleagues reported a 5-year survival rate of 30.8% after pulmonary metastectomy of metastatic breast cancer patients. However, they thought pulmonary metastectomy may not be the primary therapeutic option for metastatic breast cancer patients and they should be treated principally with chemotherapy [14]. In this study, for patients with PMBC, the mean OS and DFS time were 70.48 ± 5.98 and 63.57 ± 6.47 months, respectively. And the 5-year OS and DFS rate was 72.8% and 59.2%. Compared with previous reports, the surgical outcomes for patients with isolated PMBC were much favorable. Besides, this study has a larger number of cases than previous studies. Thus, surgical resection for isolated pulmonary metastasis from breast cancer was approved.

It is generally accepted that patients with PLC is likely have synchronous disease. However, our study showed the average DFI in the PLC group was 1.50 ± 0.5 months instead of 0. The major reason account for this result is that no all the patients received chest computed tomography (CT) scan before operation for breast cancer. Considering the high incidence rate of PLC and MBC in breast cancer patients, it is suggested to carry out routine chest CT scan for these patients.

Pathologic diagnosis is essential for pulmonary nodules that appear in patients with breast cancer, since surgical outcomes for patients with primary lung cancer and pulmonary metastases after metastectomy are significantly different. Histologic analysis including morphology and immunohistochemical staining against TTF-1 is necessary for differentiation between PLC and MBC [3]. Preoperative CT-guided needle aspiration biopsy has been established as a useful diagnostic modality for pulmonary nodules. However, fine-needle biopsy would be difficult if pulmonary nodules were small. When nonsurgical diagnosis such as fine-needle biopsy fails to reveal a pathological diagnosis, surgery, especially video-assisted thoracic surgery should be considered as an option for the diagnosis. Preoperative needle aspiration biopsy had not been performed in any patients of the present study, because the mean diameter of pulmonary nodules was relatively small (1.63 ± 0.57 cm). All patients received surgical resection for pulmonary nodules not only for diagnosis but also for the goal of treatment. Our results indicated that surgery was essential for patients with primary lung cancer after breast cancer. For patients with isolated pulmonary metastasis from breast cancer, surgical resection was approved as well. However, further controlled studies comparing surgery and systemic chemotherapy for breast cancer patients with pulmonary metastasis were necessary.

Compared with limited resection, lobectomy may lead to worse spirometry, which is not recommended for metastatic nodules [2]. However, there were still 6 patients with MBC received lobectomy in this study. The main reason for this phenomenon is that the lesions are too close to the hilum which limited resection is difficult to perform. Radiofrequency ablation (RFA) and microwave ablation (MWA) now are widely used in treating metastatic nodules with satisfactory results in preventing long term progression and local recurrences [15]. Due to the close proximity to the vessels a significant heat-sink effect was to expect in RFA, which is less evident in MWA. So under this circumstance, MWA seems to be a good alternative to lobectomy. A Further study with a large sample size is warranted to compare the advantage and disadvantage between MWA and lobectomy.

## Conclusions

Surgical outcomes of isolated pulmonary nodules in patients with breast cancer were favorable. Surgery should be considered as an option not only for the diagnosis but also for the treatment for breast cancer patients with isolated pulmonary nodules.

## Data Availability

The data used in this study are available from the corresponding author upon reasonable request.

## Abbreviations

PLC: Primary lung cancer
MBC: Metastatic breast cancer
OS: Overall survival
DFS: Disease free survival
CT: Computed tomography
PET: Positron emission tomography
TTF-1: Thyroid transcription factor-1
DFI: Disease-free Interval
NSCLCs: Non-small-cell lung cancers
RFA: Radiofrequency ablation
MWA: Microwave ablation
